# A data completion method for identifying pollution intrusion in aquifers

**DOI:** 10.1038/s41598-022-20131-9

**Published:** 2022-09-28

**Authors:** Lamia Guellouz, Faten Khayat

**Affiliations:** 1 LMHE-ENIT-Université Tunis El Manar, BP 37, 1002 Tunis Le Belvédère, Tunisia; 2 LAMSIN-ENIT-Université Tunis El Manar, Tunis, Tunisia

**Keywords:** Environmental sciences, Hydrology, Engineering, Mathematics and computing

## Abstract

The data completion method combined with the modeling of water flow and solute transport in saturated porous media is used twice to identify the extent of polluted water intrusion from the downstream boundary of an aquifer. The aim of this work is to solve the problem even though data are missing on some of the boundaries, by exploiting the fact that there are over specified data on the other boundaries. Indeed, it is assumed that the flow and the water head are known on the upstream boundary while the pollutant concentrations are known on the downstream boundary. The method developed in this work allows to determine the flows, water heads and pollutant concentrations in the whole domain. The method is applied in different aquifer configurations with pumping wells, the results are satisfactory. The model developed has shown its effectiveness in detecting the intrusion of polluted water and can be used in real cases.

## Introduction

Groundwater is often the only resource available in arid and semi-arid areas. These resources have long been considered protected by the overlying soil layers and the self-cleansing power of the soil^1^. Due to the intensification of industrial, agricultural and mining activities, and the large number of resulting by-products, many aquifers are experiencing deterioration in water quality. The presence of undesirable substances in that water makes it unfit for consumption as drinking water or for use in irrigation etc.^1^. The pollutants are of various natures: pathogenic organisms, chemical substances dissolved in water, and organic contaminants, mainly hydrocarbons that form a non-aqueous liquid phase. Pollution, most often of anthropogenic origin, comes from the spilling or spreading of polluting products on the surface of the soil, or their injection into wells or their burial in the soil^2^. These products migrate until they reach the water table. The aquifer is said to be polluted if the concentrations of these contaminants in the water exceed certain limits set by practical standards. In coastal aquifers, marine intrusion is one of the main causes of water quality degradation through salinization. Indeed, coastal areas concentrate most human activities, and aquifers are overexploited. The consequence of marine intrusion is the mixing of aquifer water with seawater, the former becomes saline and wells must be abandoned^3^. The degradation of water quality frequently concerns a particular zone of the aquifer domain and it is important to specify this zone and to prohibit any exploitation there. The delimitation of polluted areas of an aquifer is first and foremost a matter of public health and environmental protection. It is also a prerequisite for any remediation or clean-up and restoration operation. The processes of groundwater contamination by pollutant-laden water or by marine intrusion are essentially due to convection induced by the difference in density between fresh water and polluted water or seawater. The physical and numerical study of this phenomenon must therefore be carried out by considering the vertical extension of the domain in 2D or even 3D^4^. However, in the context of a large-scale regional study, it may be useful to, first, carry out a horizontal study in 2D to delineate, in a first iteration, the extension of the contamination of the aquifer and thus target the affected areas. Then, in these areas, a study involving density convection processes, can be performed in 3D or on a vertical section in order to better specify the extent of contamination^5,6^.

Consider an aquifer adjacent to a polluted area, which may be a polluted lake, a landfill, a sewage discharge, another aquifer contaminated by nitrates, heavy metals or marine intrusion; it would be wise to be able to give instructions for the use of this aquifer by rationing the pumping rates of wells to avoid contamination. Numerical modeling is a useful tool for studying such problem, however, scientists and especially modelers are in many cases confronted with missing data, such as aquifer delineation, hydrodynamic characteristics, upstream and downstream flow conditions, salt or pollutant transport parameters, salt or pollutant concentrations at boundaries. Inverse problem solving is used to identify missing data. Many studies are performed in hydrogeology using these methods. Comprehensive and exhaustive reviews of the use of inverse problems in hydrogeology are available such as those by De Marsily et al.^7^ and Zhou et al.^8^. Many papers deal with parameter identification^9–11^, while others focus on determining boundary conditions and positions and flows of wells^12–14^.

The objective of this work is to develop a numerical model to study and quantify the pollution of an aquifer, exploited by pumping wells, and located upstream of a polluted area. In this case, the direction of the flow, from or towards this zone, is dependent on the pumping rates of the wells. In the case of pollution intrusion, the extent of the pollution should be determined. Since not all boundary conditions of this aquifer are known a priori, the data completion method is used to solve the problem. This method has been applied in several fields, including medical diagnosis, remote sensing, traffic studies, and groundwater flow^15^. The data completion method has also been used for marine intrusion problems in^6,15,16^. In^6^, the authors applied this technique to locate the land-sea interface in a coastal aquifer. In^16^, this method was used by Mansouri et al. to identify the well position and pumping rate from the knowledge of the hydraulic head and flow on a part of the domain boundary. In^15^, Bel Hadj Hassine et al. have reconstructed the fluid flow and the concentration of a solute on a non-accessible part of the boundary for the advection-diffusion equations. In all these studies, only stationary flow problems were considered. In this work, two coupled problems are dealt with: the flow of water in the aquifer and the transport of the pollutant as a solute. Boundary conditions can be imposed pressures, flow rates or solute concentrations. One or more of these conditions may be known on one boundary and missing on another. The inverse algorithm, based on data completion, determines the missing values and thus solves the problem. The novelty of this work lies in the resolution of two coupled problems of flow and transport, which are also non-stationary. The partial differential equations of the two direct problems are solved by the finite element method. The model is tested and used to identify pollution intrusion in an aquifer due to a high pumping rate. The direction and intensity of the exchange flow of the aquifer with the polluted zone are determined and, if pollution intrusion occurs, the concentrations of the pollutant in the aquifer are calculated. The originality of the application case is that the pollution comes from a neighboring aquifer, known a priori to be polluted and which is located downstream of the studied area. This domain can be for example the water table under a spreading or discharge area, or under an agricultural perimeter which is excessively fertilized or treated with pesticides... The existence of pumping wells in the aquifer under study means that the direction of flow can be reversed and polluted water from downstream can enter the aquifer: this is why it is called pollution intrusion. This is a relatively frequent situation in arid zones in relation to the overexploitation of aquifers following the increasing water demand^17^.

## Problem statement

The study and monitoring of an aquifer threatened by pollution intrusion requires knowledge of the geometry of the aquifer, its hydrodynamic characteristics such as transmissivity and porosity, and the diffusion-dispersion parameters of the pollutant propagating as a solute. Upstream and downstream water flows, water heads and pollutant concentrations on the boundaries must be determined. Some values of these physical quantities are measured or determined by previous studies and others must be calculated.

**Figure 1:**
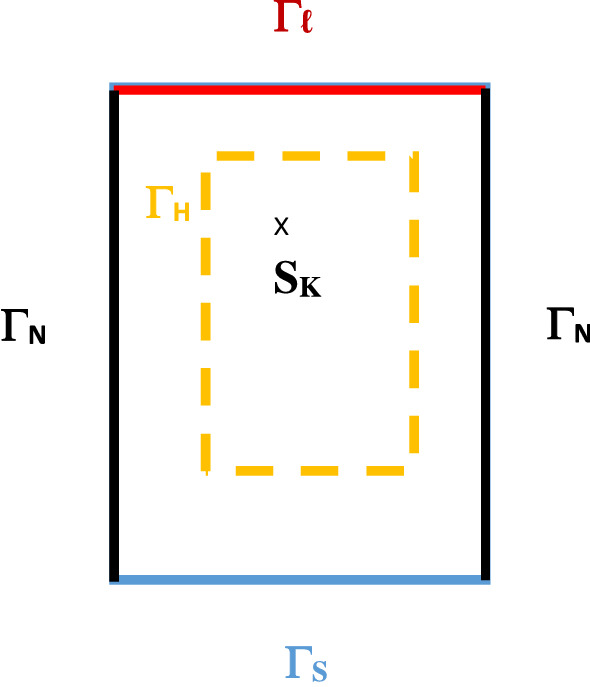
Domain configuration.

To study the problem of pollution intrusion in an aquifer, and as a primary approach, a theoretical case with a simple geometry and constant flow and transport parameters is considered. The unconfined aquifer is a rectangular domain (Fig. 1), it is bordered by a polluted zone on the lower side $\Gamma _s$ and the upper side $\Gamma _{\ell }$ can be the upstream boundary of the aquifer or a line of piezometers or homogeneous flow. A pumping well is located at point $S_k$. The flow regime in the aquifer is the primary factor governing pollution intrusion. Pollution intrusion occurs when the flow of water from the aquifer into the polluted zone decreases, causing the contaminated water to advance into the aquifer.

The mixing zone between contaminated and clean water, where density-induced flow occurs at high concentrations, is called the transition zone. This zone may be wide, narrow, or approximated by a sharp interface, depending on the characteristics of the aquifer and the nature of the pollutant. A large-scale domain is adopted in this work. By comparison, the mixing zone is narrow. The trace pollutant is dissolved in water, so the density effect is not taken into account. The flow in the aquifer is essentially horizontal according to the conception of underground hydrodynamics under the assumptions of Dupuit^2,18^. Polluted water and fresh water are considered as one single liquid phase with different concentrations of pollutant as solute carried by the water. The solute transport processes taken into account are convection, diffusion and dispersion. Therefore, two coupled problems are solved: water flow and solute transport in porous media. They are described by unsteady partial differential equations, and need initial conditions and boundary conditions (see Eqs. (1) and (2) below). Under natural conditions, the upstream boundary of the aquifer is $\Gamma _{\ell }$ and the downstream boundary is $\Gamma _s$. Anthropogenic action: high well pumping rate, leads to pollution intrusion by reversing the direction of flow. The $\Gamma _{\ell }$ boundary is, a priori, a hydrodynamically well defined. The hydraulic head can be measured by a series of piezometers. The flow rate, perpendicular to $\Gamma _{\ell }$, can be determined according to the Dupuit hypothesis, by hydraulic head measurements given by two rows of piezometers separated by a few meters. It can also be the  result of previous studies, or it can be a well known aquifer boundary such as the discharge of an upstream aquifer. It is also assumed that this area is beyond the influence of the contaminated water intrusion. On $\Gamma _s$, the polluted side boundary, the hydraulic head and water flow are unknown and variable. The flow can be either towards the polluted area (discharge case) or towards the aquifer (intrusion situation). This boundary can also be in a mixed situation i.e. partly discharge and partly intrusion. Since the water flow is essentially parallel to $\Gamma _N$, these lateral boundaries are zero flow. The boundary conditions of the solute transport problem are variable over $\Gamma _s$: in intrusion the pollutant concentration is that of the polluted zone and in discharge that of the aquifer. In $\Gamma _{\ell }$ the pollutant concentration is that of the freshwater entering upstream of the aquifer, i.e., very low and near zero. Since there is over-specified boundary conditions in $\Gamma _{\ell }$ (both water head and flow) while missing in $\Gamma _s$ for the flow equation, the Data Completion method is used to solve the problem. On the other hand, when pollution intrusion is proved, the opposite situation occurs for the transport problem: boundary conditions are over-specified in $\Gamma _s$ while they are missing in $\Gamma _{\ell }$. Thus, the Data Completion method is used twice to solve the pollution intrusion problem. In the next section, the coupled problems of flow and solute transport in an aquifer are presented and the application of Data Completion method to these problems is detailed.

## Data completion

For $\Omega \subset {\mathbb R}^2$ a lipschitz bounded and connected domain such that $\partial \Omega$ is composed of three disjoint parts of non-vanishing measure: $\Gamma _s$ is the polluted side, $\Gamma _N$ is the lateral sides and $\Gamma _{\ell }$ corresponds to the upstream side. Consider the problem of pollutant transport as a dissolved solute in the aquifer water, at a concentration C:

1 $$\begin{aligned} ({{\mathcal {P}}}{{\mathcal {I}}})\,\,\left\{ \begin{array}{lll} \displaystyle \frac{\partial C}{\partial t}-div(D \nabla C- V C) = \displaystyle \sum _{k=1}^{2} Q_{ck} \delta (x-S_k) &{} \text{ in } \Omega \,\times \,\,[t_0,t_f] \\ (D \nabla C) \cdot n =0 &{} \text{ on } \Gamma _N \,\times \,\,[t_0,t_f]\\ (D \nabla C) \cdot n =\Phi _c &{} \text{ on } \Gamma _c \,\times \,\,[t_0,t_f]\\ C=\Psi _c &{} \text{ on } \Gamma _c \,\times \,\,[t_0,t_f] \\ C(x,y,t_0)=C_0(x,y) &{} \text{ in } \Omega \end{array} \right. \end{aligned}$$

$\Phi _c$ and $\Psi _c$ are the overspecified data corresponding respectively to the prescribed flux and concentration on the accessible part of the boundary $\Gamma _c$ on which measures are available. *D* is the hydrodynamic diffusion-dispersion second-order tensor and *V* is the Darcy velocity. We make the assumption that the matrix *D* is symmetric positive definite and that $V \cdot n >0$ on $\Gamma _c$. For $k=1,2$, $Q_{ck}$ is the well abstraction corresponding to the $k^{th}$ point source $S_k$ whose coordinates are $(x_k,y_k$). $\delta$ denotes the Dirac distribution.

We denote by $H^{1}(\Omega )=\left\{ {\varvec{u}}\in L^{2}(\Omega ),\,\partial {\varvec{u}}_{i}/\partial x_{j}\in L^{2}(\Omega )\, \text{ for } \,i,j=1,2 \right\}$ the standard Sobolev space equipped with usual first-order Sobolev norm. For any portion $\Gamma$ of the boundary $\partial \Omega$, we note $H^{1/2}(\Gamma )$ the space of the traces over $\Gamma$ of elements of $H^{1}(\Omega )$. We designate by ${{\mathcal {V}}}^{1}(\Gamma )=H_{00}^{1/2}(\Gamma )$ the set of all the restrictions to $\Gamma$ of the functions of $H^{1/2}(\partial \Omega )$ that vanish on $\partial \Omega \setminus \Gamma$. It’s topological dual space will be noted ${{\mathcal {V}}}^{-1}(\Gamma )$.

By the Darcy law, the velocity *V* satisfies the relation:

$$\begin{aligned} V=-T grad(h) \end{aligned}$$

where *h* is the hydraulic head, obtained as a solution of the flow problem in saturated porous media:

2 $$\begin{aligned} ({{\mathcal {P}}}{{\mathcal {I}}}^H)\left\{ \begin{array}{lll} \displaystyle \frac{\partial h}{\partial t}-div(T \nabla h) = \displaystyle \sum _{k=1}^{2} Q_{k} \delta (x-S_k) &{} \text{ in } \Omega \,\times \,\,[t_0,t_f]\\ T \nabla h \cdot n =0 &{} \text{ on } \Gamma _N \,\times \,\,[t_0,t_f]\\ T \nabla h \cdot n =\bar{H_c} &{} \text{ on } \Gamma _c \,\times \,\,[t_0,t_f]\\ h=\bar{h_c} &{} \text{ on } \Gamma _c \,\times \,\,[t_0,t_f] \\ h(x,y,t_0)=0 &{} \text{ in } \Omega \end{array} \right. \end{aligned}$$

here *T* is the transmissivity field, $\bar{H_c}$ and $\bar{h_c}$ are respectively the prescribed flux and head on $\Gamma _c$.

First, we suppose that the hydraulic head is unknown on the polluted side, so for the inverse problem $({{\mathcal {P}}}{{\mathcal {I}}}^H)$, $\Gamma _i=\Gamma _s$ and $\Gamma _c=\Gamma _{\ell }$. For the pollutant transport inverse problem, the concentration *C* is wellknown at the polluted side, which is the $\Gamma _c$ for $({{\mathcal {P}}}{{\mathcal {I}}})$ and unknown at the upstream side. So we will use the reconstructed hydraulic head on the polluted side and the Darcy law to reconstruct concentration on $\Gamma _i=\Gamma _{\ell }$.

In order to reconstruct the missing hydraulic head and concentration on the corresponding $\Gamma _i$, we use a method developped by Andrieux et al.^19^. We first introduce a fictious inner boundary $\Gamma _F$ excluding the region containing the wells. Thus we obtain a fictious domain $\Omega _F$ such that $\partial \Omega _F=\Gamma _N \cup \Gamma _c \cup \Gamma _i\cup \Gamma _F$. In the sequel, we note $\Gamma _H= \Gamma _i \cup \Gamma _F$ the new inaccessible boundary on which we must reconstruct the data. The Cauchy problem consists then in finding $(t,\varphi )$ such that:

3 $$\begin{aligned} \left\{ \begin{array}{lll} \displaystyle \frac{\partial C}{\partial t}-div(D \nabla C- V C) =0 &{} \text{ in } \Omega _F \,\times \,\,[t_0,t_f] \\ (D \nabla C) \cdot n =0 &{} \text{ on } \Gamma _N \,\times \,\,[t_0,t_f]\\ (D \nabla C) \cdot n =\Phi _c, \,\, C=\Psi _c &{} \text{ on } \Gamma _c \,\times \,\,[t_0,t_f] \\ (D \nabla C) \cdot n =\varphi , \,\, C=t &{} \text{ on } \Gamma _H \,\times \,\,[t_0,t_f] \\ C(x,y,t_0)=C_0(x,y) &{} \text{ in } \Omega _F \end{array} \right. \end{aligned}$$

Then, we split each one of the problems (2) and (3) into two mixed and well-posed problems which use simultaneously the overspecified data: the first one uses the Dirichlet condition on $\Gamma _c$ as given data and called Dirichlet problem ($P_D$), the second one uses the Neumann condition as given data on $\Gamma _c$ and consequently called Neumann problem ($P_N$):

4 $$\begin{aligned}&(P_{D}^H)\left\{ \begin{array}{ll} \displaystyle \frac{\partial h_1}{\partial t}-div(T \nabla h_1)= 0 &{} \text{ in } \Omega _F\,\times \,\,[t_0,t_f] \\ (T \nabla h_1) \cdot n =0 &{} \text{ on } \Gamma _N \,\times \,\,[t_0,t_f]\\ h_{1}=\bar{h_c} &{} \text{ on } \Gamma _c \,\times \,\,[t_0,t_f] \\ (T \nabla h_1) \cdot n =\lambda ^h &{} \text{ on } \Gamma _H \,\times \,\,[t_0,t_f] \\ h_1(t_0)=h_0 &{} \text{ in } \Omega _F \end{array} \right. \end{aligned}$$

5 $$\begin{aligned}&(P_{N}^H) \left\{ \begin{array}{ll} \displaystyle \frac{\partial h_2}{\partial t}-div(T \nabla h_2) = 0&{} \text{ in } \Omega _F \,\times \,\,[t_0,t_f]\\ (T \nabla h_2) \cdot n =0 &{} \text{ on } \Gamma _N \,\times \,\,[t_0,t_f]\\ (T \nabla h_2) \cdot n =\bar{H_c} &{} \text{ on } \Gamma _c \,\times \,\,[t_0,t_f]\\ h_{2} = \tau ^h &{} \text{ on } \Gamma _H \,\times \,\,[t_0,t_f] \\ h_2(t_0)=h_0 &{} \text{ in } \Omega _F \end{array} \right. \end{aligned}$$

We do the same thing for (3):

6 $$\begin{aligned}&(P_{D}^C)\left\{ \begin{array}{ll} \displaystyle \frac{\partial C_1}{\partial t}-div(D \nabla C_1- V C_1)= 0 &{} \text{ in } \Omega _F\,\times \,\,[t_0,t_f] \\ (D \nabla C_1) \cdot n =0 &{} \text{ on } \Gamma _N \,\times \,\,[t_0,t_f]\\ C_{1}=\Psi _c &{} \text{ on } \Gamma _c \,\times \,\,[t_0,t_f] \\ (D \nabla C_1) \cdot n =\lambda &{} \text{ on } \Gamma _H \,\times \,\,[t_0,t_f] \\ C_1(t_0)=C_0 &{} \text{ in } \Omega _F \end{array} \right. \end{aligned}$$

7 $$\begin{aligned}&(P_{N}^C) \left\{ \begin{array}{ll} \displaystyle \frac{\partial C_2}{\partial t}-div(D \nabla C_2- V C_2) = 0&{} \text{ in } \Omega _F \,\times \,\,[t_0,t_f]\\ (D \nabla C_2) \cdot n =0 &{} \text{ on } \Gamma _N \,\times \,\,[t_0,t_f]\\ (D \nabla C_2) \cdot n =\Phi _c &{} \text{ on } \Gamma _c \,\times \,\,[t_0,t_f]\\ C_{2} = \tau &{} \text{ on } \Gamma _H \,\times \,\,[t_0,t_f] \\ C_2(t_0)=C_0 &{} \text{ in } \Omega _F \end{array} \right. \end{aligned}$$

$C_1$ and $C_2$ are equal only when the pair $(\tau ,\lambda )$ meets the real data $(t,\varphi )$ on $\Gamma _H$.

In order to make the problem “symmetric”, we will define some function changes. Since *D* is supposed to be symmetric positive definite, we define the vector *K* and the positive constant $\varrho$ such that:

$$\begin{aligned} 2D K-V=0 \text{ and } \varrho =K\cdot DK. \end{aligned}$$

Now, for $X=(x,y)\in \Omega _F$, we introduce new functions^20^: $U_1=e^{- K .X} C_1$ and $U_2=e^{- K .X} C_2$. These functions are solutions of the systems:

8 $$\begin{aligned}&(P_{D}^{New})\left\{ \begin{array}{ll} \displaystyle \frac{\partial U_1}{\partial t}-div(D \nabla U_1) + \varrho U_1= 0 &{} \text{ in } \Omega _F\,\times \,\,[t_0,t_f] \\ D\nabla U_1 \cdot n + \frac{1}{2} V \cdot U_1 \cdot n = 0 &{} \text{ on } \Gamma _N \,\times \,\,[t_0,t_f]\\ U_{1}= \overline{\Psi _c} &{} \text{ on } \Gamma _c \,\times \,\,[t_0,t_f] \\ D\nabla U_1 \cdot n + \frac{1}{2} V \cdot U_1 \cdot n =\Lambda &{} \text{ on } \Gamma _H \,\times \,\,[t_0,t_f]\\ U_1(t_0)=\overline{C_0} &{} \text{ in } \Omega _F \end{array} \right. \end{aligned}$$

9 $$\begin{aligned}&(P_{N}^{New}) \left\{ \begin{array}{ll} \displaystyle \frac{\partial U_2}{\partial t}-div(D \nabla U_2)+ \varrho U_2 = 0&{} \text{ in } \Omega _F \,\times \,\,[t_0,t_f]\\ D\nabla U_2 \cdot n + \frac{1}{2} V \cdot U_2 \cdot n = 0 &{} \text{ on } \Gamma _N \,\times \,\,[t_0,t_f]\\ D\nabla U_2 \cdot n + \frac{1}{2} V \cdot U_2 \cdot n =\overline{\Phi _c} &{} \text{ on } \Gamma _c \,\times \,\,[t_0,t_f]\\ U_{2} = \Theta &{} \text{ on } \Gamma _H \,\times \,\,[t_0,t_f]\\ U_2(t_0)=\overline{C_0} &{} \text{ in } \Omega _F \end{array} \right. \end{aligned}$$

where we have set

$$\begin{aligned} \overline{\Psi _c}&= e^{- K .X}\Psi _c, \qquad&\overline{\Phi _c}&= e^{- K .X}\Phi _c, \qquad&\overline{C_0}&=e^{- K .X} C_0\\ \Lambda&= e^{- K .X}\lambda , \qquad&\Theta&= e^{- K .X}\tau .&\\ \end{aligned}$$

So, let us define the functional

$${\mathcal{E}}(\Lambda ,\Theta ) = \frac{1}{2}\int\limits_{{t_{0} }}^{{t_{f} }} {} \int\limits_{\Omega } {\left( {|(D^{{1/2}} \nabla U_{1} - D^{{1/2}} \nabla U_{2} |^{2} + {\varrho }|U_{1} - U_{2} |^{2} } \right)dx + \frac{1}{4}} \int\limits_{{t_{0} }}^{{t_{f} }} {\int\limits_{{\Gamma _{c} \cup \Gamma _{N} }} {(V \cdot n)|U_{1} - U_{2} |^{2} } }$$

### *Remark 1*

1. $U_1$ and $U_2$ are equal only when the pair $(\Lambda ,\Theta )$ meets the real data $(e^{- K .X}\varphi , e^{- K .X}t)$ on $\Gamma _H$ which is equivalent to say that $(\lambda ,\tau )$ meets the real data $(\varphi , t)$ on $\Gamma _H$.
2. $\mathcal {E}(\Lambda ,\Theta )$ reaches its minimum when $U_1=U_2=U$ with $U=e^{- K .X}C$ and where *C* is the unique solution to the data completion problem.
3. The inverse problem (3) is equivalent to the following minimization:
  10 $$\begin{aligned} \begin{array}{r} (\Pi , \Upsilon )=\underset{{(\Lambda , \Theta )}}{{arg\,min}}\,\, \mathcal {E}(\Lambda ,\Theta ), \end{array} \end{aligned}$$
  with $$(\Pi , \Upsilon )=(e^{- K .X}\varphi , e^{- K .X}t)$$, $$\Theta \in {{\mathcal {V}}}^{1}(\Gamma _H)$$ and $$\Lambda \in {{\mathcal {V}}}^{-1}(\Gamma _H)$$.
4. $\mathcal {E}$ is an energy-like error functional that represents the energy gap between the so-called Neumann solution of (9) and the Dirichlet solution of (8) (see^13,21^).

### **Theorem 2**

1. *The functional* $(\Lambda , \Theta )\longmapsto \mathcal {E}(\Lambda , \Theta )$ *is a positive quadratic functional. It’s strictly convex on* ${{\mathcal {V}}}^{-1}(\Gamma _H) \times {{\mathcal {V}}}^{1}(\Gamma _H)$ *and consequently has a unique minimum for a compatible data* $(\overline{\Phi _c},\overline{\Psi _c})$.
2. *When* $\mathcal {E}$ *reaches its minimum, the solutions* $C_{1}$ *and* $C_{2}$ *of the inverse problem* (3) *verify the first-order optimality conditions:*
  11 $$\begin{aligned} \left\{ \begin{array}{ll} C_{1}=C_{2} + \alpha &{} \text{ on } \Gamma _H\,\times \,\,[t_0,t_f] \\ (D \nabla C_1- \frac{1}{2} V C_1) \cdot n= (D \nabla C_2- \frac{1}{2} V C_2) \cdot n &{} \text{ on } \Gamma _H \,\times \,\,[t_0,t_f] \end{array} \right. \end{aligned}$$
  *where* $\alpha$ *is a constant.*

### *Proof*

1. A similar calculus as in^15^ shows that the first derivatives of $\mathcal {E}$ are given by:
  12 $$\begin{aligned} \frac{\partial \mathcal {E}}{\partial \Lambda } \cdot w =&\displaystyle \int _{t_0}^{t_f}\displaystyle \int _{\Gamma _H} \left( D \nabla (U_1-U_2)\cdot n \right) r_1^{w}, \end{aligned}$$
  13 $$\begin{aligned} \frac{\partial \mathcal {E}}{\partial \Theta } \cdot h =&- \displaystyle \int _{t_0}^{t_f}\displaystyle \int _{\Gamma _H} (D \nabla r_2^{h} \cdot n) (U_1 -U_2) , \end{aligned}$$
  where $r_1^w$ and $r_2^h$ are respectively the solution of
  14 $$\begin{aligned} \left\{ \begin{array}{ll} \displaystyle \frac{\partial r_1^w}{\partial t}-div(D \nabla r_1^w ) + \varrho r_1^w= 0 &{} \text{ in } \Omega _F \,\times \,\,[t_0,t_f]\\ \left( D\nabla r_1^w + \frac{1}{2} V \cdot r_1^w \right) \cdot n = 0 &{} \text{ on } \Gamma _N \,\times \,\,[t_0,t_f]\\ r_1^w = 0 &{} \text{ on } \Gamma _c \,\times \,\,[t_0,t_f] \\ \left( D\nabla r_1^w + \frac{1}{2} V \cdot r_1^w \right) \cdot n =w &{} \text{ on } \Gamma _H \,\times \,\,[t_0,t_f]\\ r_1^w(t_f)=0 &{} \text{ in } \Omega _F \end{array} \right. \end{aligned}$$
  15 $$\begin{aligned} \left\{ \begin{array}{ll} \displaystyle \frac{\partial r_2^h}{\partial t}-div(D \nabla r_2^h) + \varrho r_2^h = 0&{} \text{ in } \Omega _F \,\times \,\,[t_0,t_f]\\ \left( D\nabla r_2^h + \frac{1}{2} V \cdot r_2^h \right) \cdot n = 0 &{} \text{ on } \Gamma _N \,\times \,\,[t_0,t_f]\\ \left( D\nabla r_2^h + \frac{1}{2} V \cdot r_2^h \right) \cdot n = 0 &{} \text{ on } \Gamma _c \,\times \,\,[t_0,t_f]\\ r_2^h = h &{} \text{ on } \Gamma _H \,\times \,\,[t_0,t_f] \\ r_2^h(t_f)=0 &{} \text{ in } \Omega _F \end{array} \right. \end{aligned}$$
  In the same manner, the second derivatives are
  $$\begin{aligned} \begin{array}{ll} \frac{\partial ^2 \mathcal {E}}{\partial \Lambda ^2 } (w,w)&{} = \displaystyle \int _{t_0}^{t_f}\displaystyle \int _{\Omega _F}{ D | \nabla r_1^{w}|^2}+ \displaystyle \int _{t_0}^{t_f}\displaystyle \int _{\Omega _F}{\varrho | r_1^{w}|^2 } \\ \,&{} \\ \frac{\partial ^2 \mathcal {E}}{\partial \Theta ^2 } (h,h)&{} = \displaystyle \int _{t_0}^{t_f}\displaystyle \int _{\Omega _F}{ D |\nabla r_2^{h}| ^2} + \displaystyle \int _{t_0}^{t_f}\int _{\Omega _F}{\varrho | r_2^{h}|^2 }+ \frac{1}{2} V \cdot n \displaystyle \int _{\Gamma _c}{|r_2^{h}|^2} \end{array} \end{aligned}$$
  which shows the convexity of $\mathcal {E}$. To prove the strict convexity, we use the trace theorem to deduce that we have, for some constant $c>0$,
  $$\begin{aligned} \begin{array}{ll} \frac{\partial ^2 \mathcal {E}}{\partial \Lambda ^2 } (w,w)&{} \, \geqslant \,\,c \Vert r_1^{w}\Vert ^2_{H^1(\Omega _F)} \geqslant c \Vert r_1^{w}\Vert ^2_{{{\mathcal {V}}}^{1}(\Gamma _H)} \\ \,&{} \\ \frac{\partial ^2 \mathcal {E}}{\partial \Theta ^2 } (h,h)&{} \,\, \geqslant \,\,c \Vert r_2^{h}\Vert ^2_{H^1(\Omega _F)} \geqslant c \Vert r_2^{h}\Vert ^2_{{{\mathcal {V}}}^{1}(\Gamma _H)}=c \Vert h \Vert ^2_{{{\mathcal {V}}}^{1}(\Gamma _H)} \end{array} \end{aligned}$$
2. From Eqs. (12) and (13), we deduce that when $\mathcal {E}$ reaches its minimum, the solutions $U_{1}$ and $U_{2}$ verify:
  16 $$\begin{aligned} \left\{ \begin{array}{ll} U_{1}=U_{2} + \alpha &{} \text{ on } \Gamma _H, \\ \left( D\nabla U_1\right) \cdot n = \left( D\nabla U_2\right) \cdot n &{} \text{ on } \Gamma _H \end{array} \right. \end{aligned}$$
  so by the inverse variable change, we have immediately the conditions (11).

$$\square$$

Now, we can use the above transmission conditions to set up an iterative procedure among subdomains and based on the preconditioned gradient algorithm:
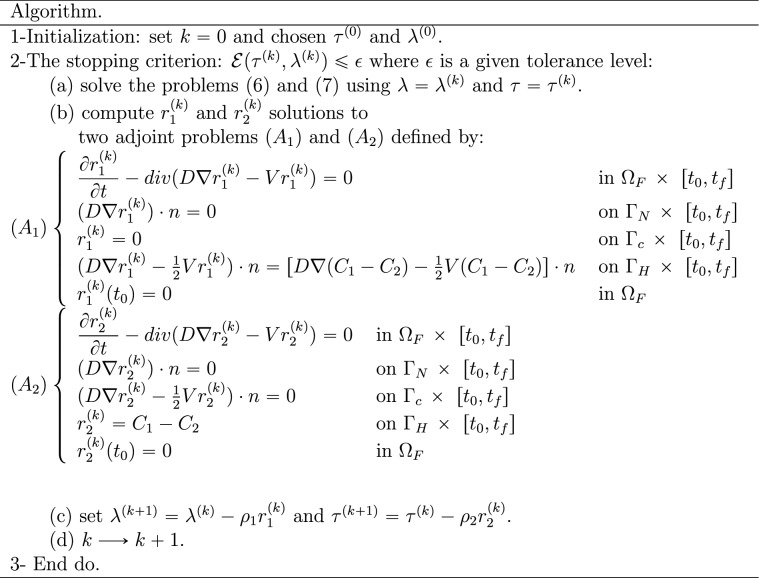
where $\rho _1$ and $\rho _2$ are the relaxation parameters.

## Numerical results

We consider a rectangular domain with edges $\ell _1=5 \; \text{km}$ and $\ell _2=7\; \text{km}$. The upstream side is the upper edge refered to by $\Gamma _{\ell }=[0,5]\times \{7\}$, the polluted side is the lowest one and denoted by $\Gamma _s =[0,5]\times \{0\}$ as depicted in Fig. 1. The lateral edges with zero flux correspond to $\Gamma _N$. $\Gamma _H$ is chosen to be the rectangle: $[1.5,3.5]\times [2,5]$. The three computed examples differ by the location and the intensity of the points sources. The transmissivity is taken $T=0.1\, \text{m}^2\text{s}^{-1}$ for the first and the second examples while it is $T=0.0001\, \text{m}^2\text{s}^{-1}$ for the third one. *D* is the diagonal matrix such that $d_{11}=d_{22}=10^{-6}$. Synthetic data are generated with constant exact values: $h=3$ on $\Gamma _{\ell }$ and $h=1$ on $\Gamma _s$ for the hydraulic problem corresponding to both first and second examples. For the last one, we took $h=0$ on $\Gamma _s$. For the transport problem, *C* is chosen to be constant on each boundary such that: $C=30$ on $\Gamma _s$ and $C=1$ on $\Gamma _{\ell }$. The stopping criterion is $\varepsilon = 0.015$. We chose $t_0=0$ and $t_f=3$ for first and second examples and $t_f=2$ for the third one. We initialize with a constant concentration: $C_0=C(t=0)=1$ on $\Omega$ for all cases. All calculations are run under the Freefem Software environnement^22^. For time treatment, a finite difference method and precisely the Euler scheme scheme is used. The computation time was for case 1: 417.187 s, for case 2: 211.392 s and for case 3: 238.163 s.

### First example

This first example referred to in the figures by *Case 1* corresponds to two point sources such that $S_1(2,2.8)$ with $Q_1=-0.1$ and $S_2(2.8,3.5)$ with $Q_2=-0.8$.

**Figure 2:**
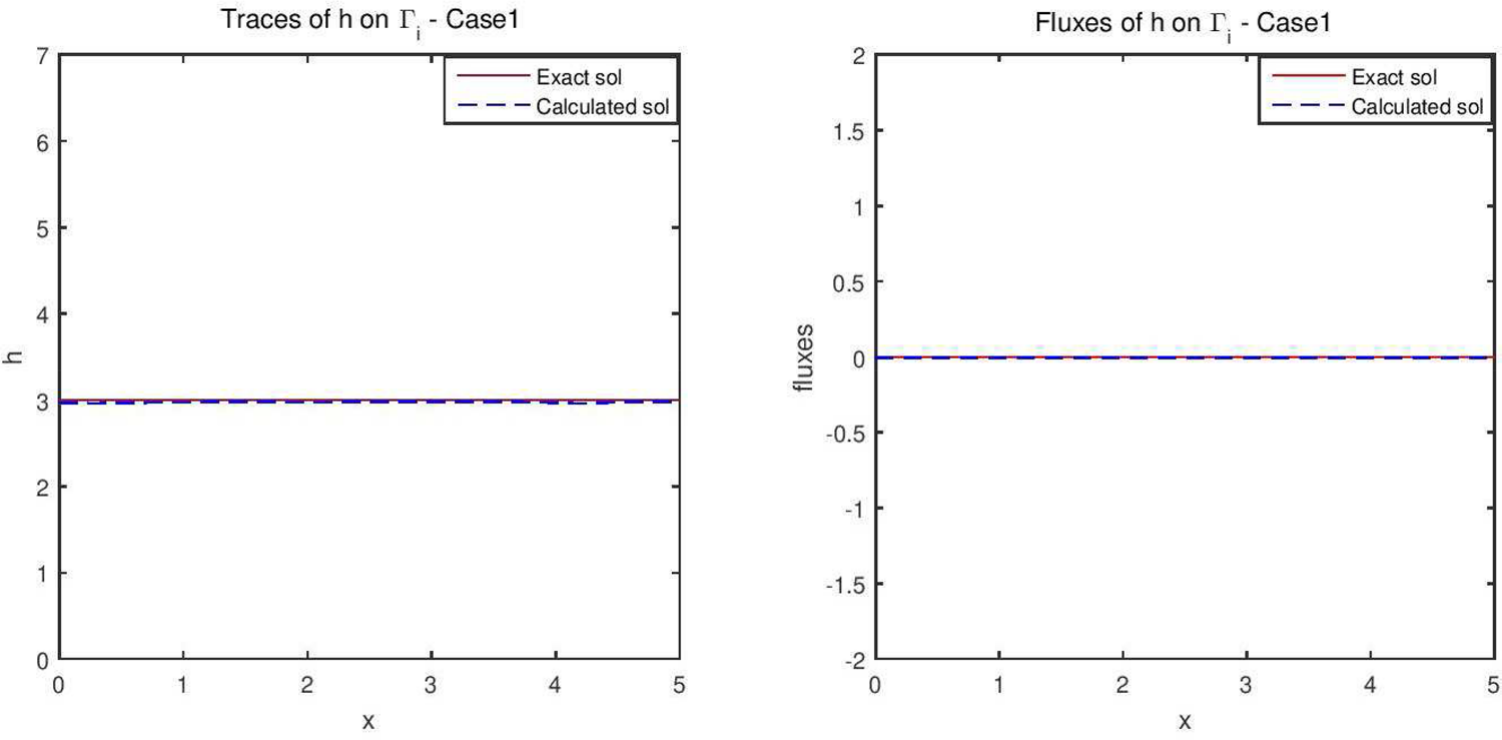
The reconstructed flow—Case 1.

**Figure 3:**
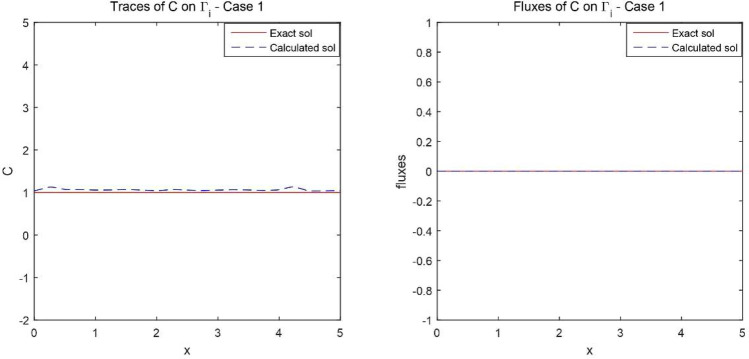
The reconstructed concentration—Case 1.

**Figure 4:**
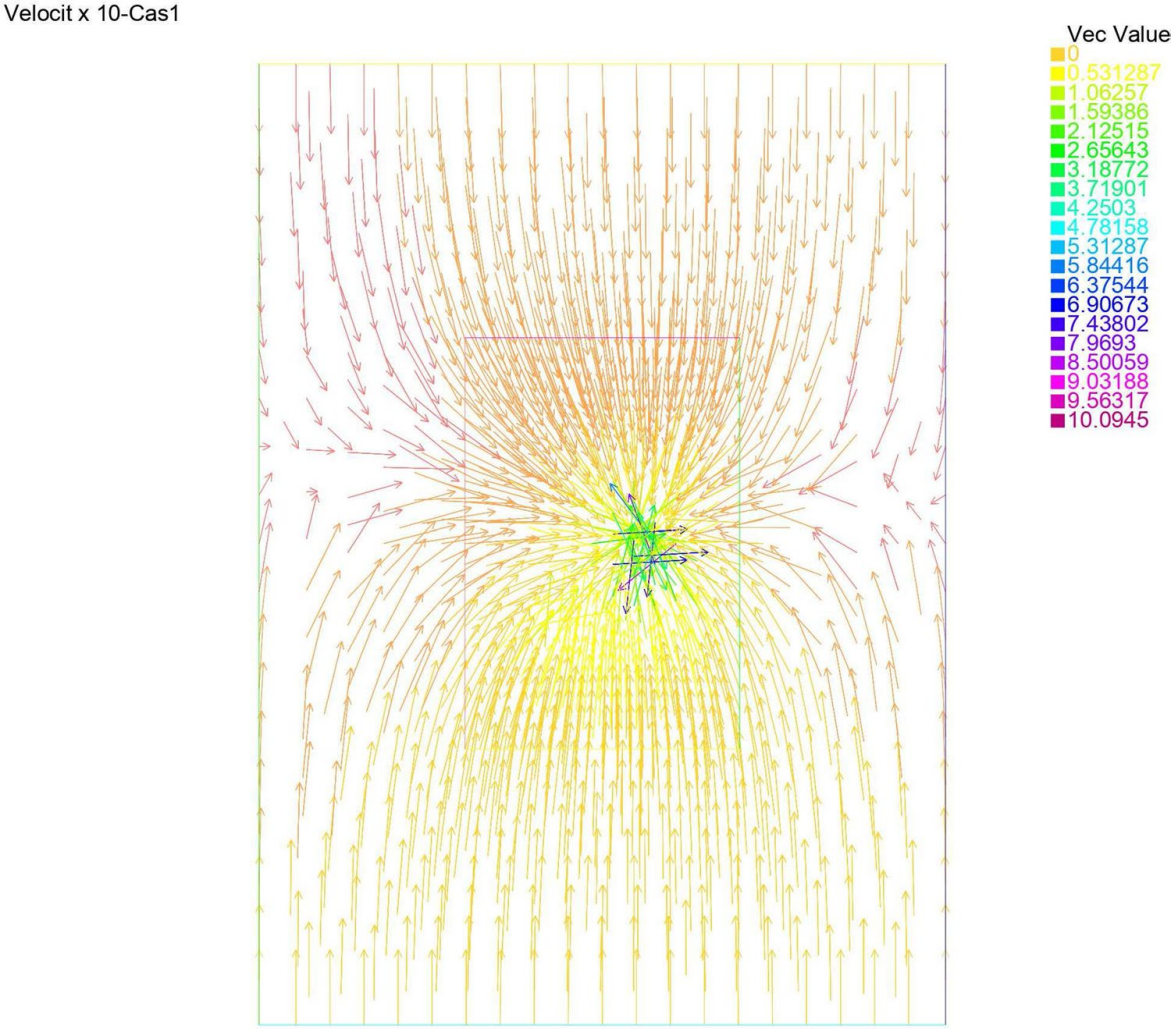
Velocity x 10—Case 1.

Figures 2 and 3 give the reconstructed traces and fluxes for the flow and the transport problem respectively. Figure 4 gives the flow velocity. Since the values are small, they are multiplied by 10 to represent them correctly. In Fig. 11 are shown the isovalues of the pollutant concentrations in the aquifer at the final time.

### Second example

The second example denoted by *Case 2* corresponds to the same coordinates for the point source as in Case 1, but with $Q_1=-1$ and $Q_2=-0.02$.

**Figure 5:**
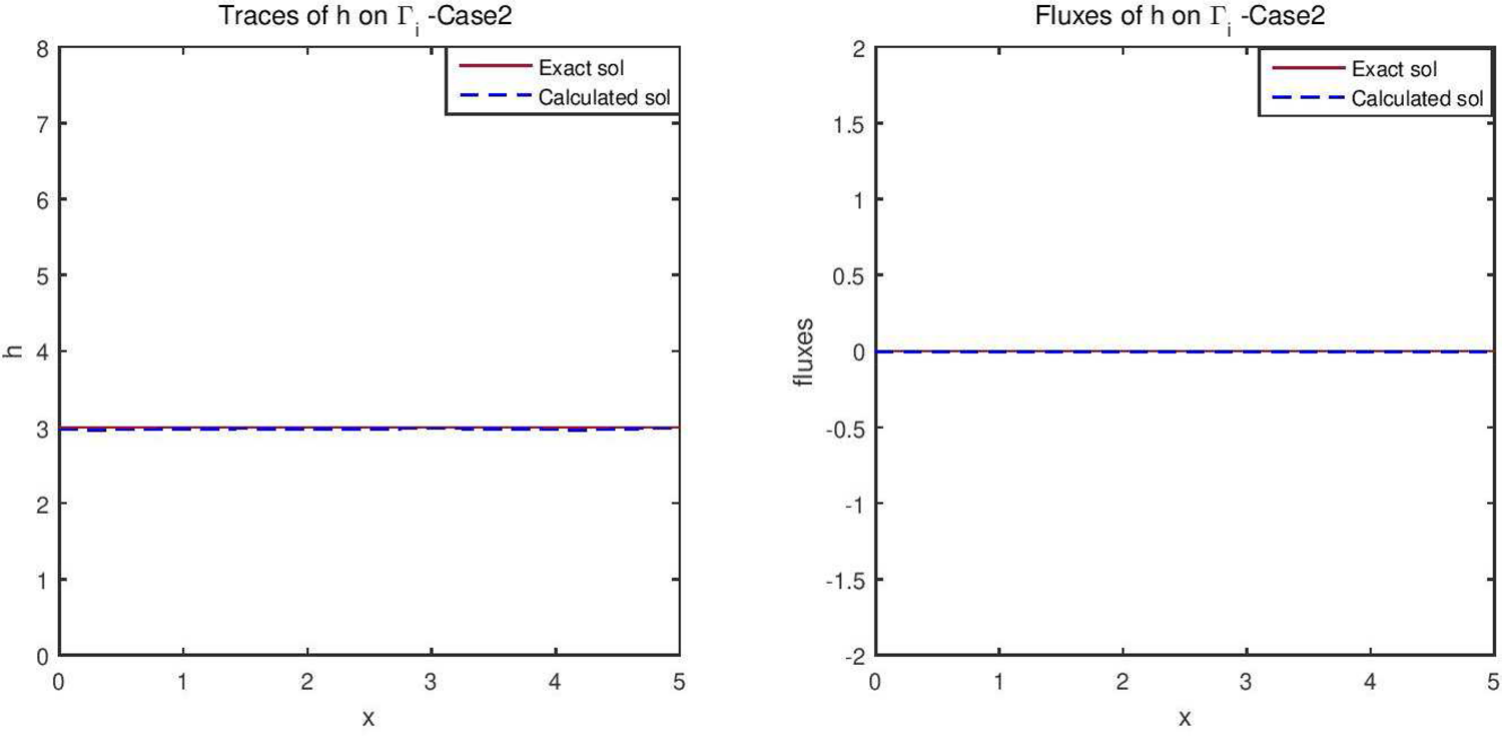
The reconstructed flow—Case 2.

**Figure 6:**
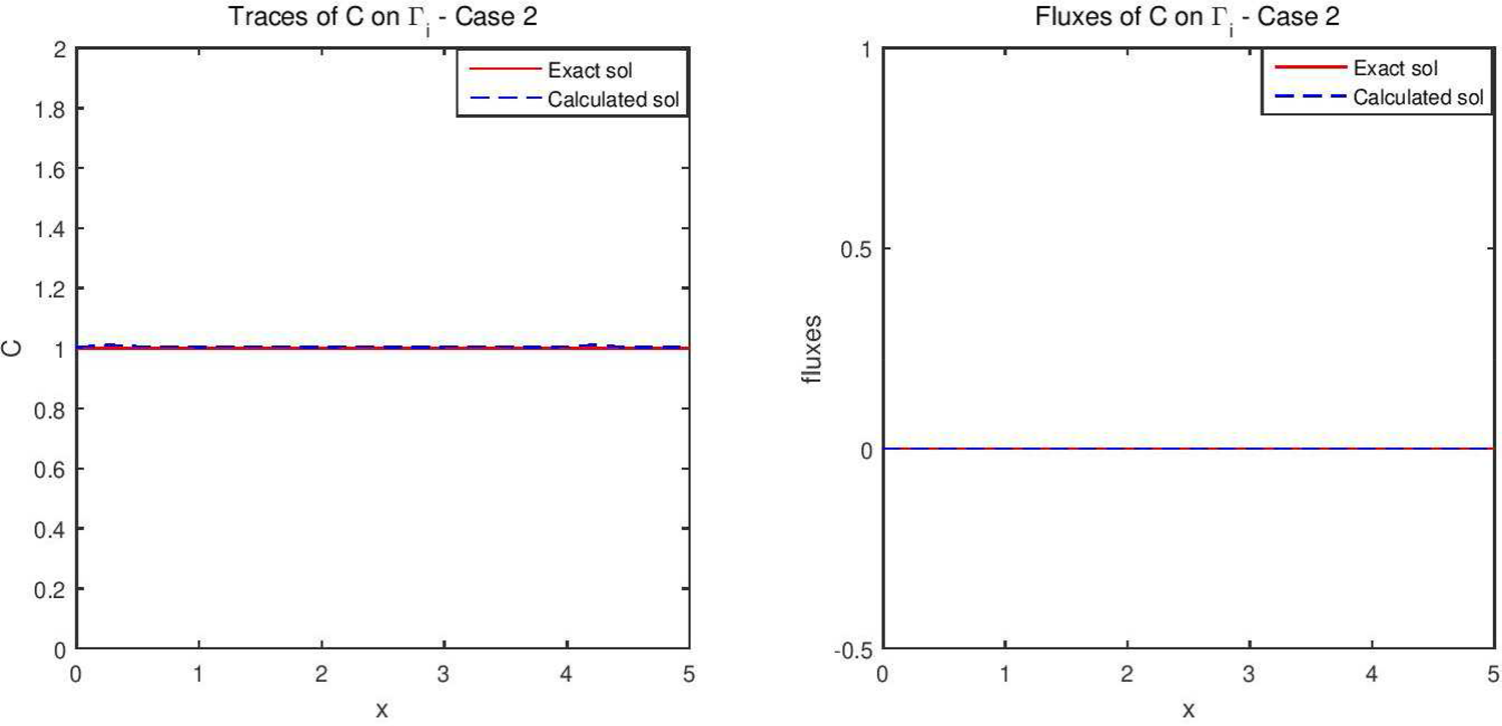
The reconstructed concentration—Case 2.

**Figure 7:**
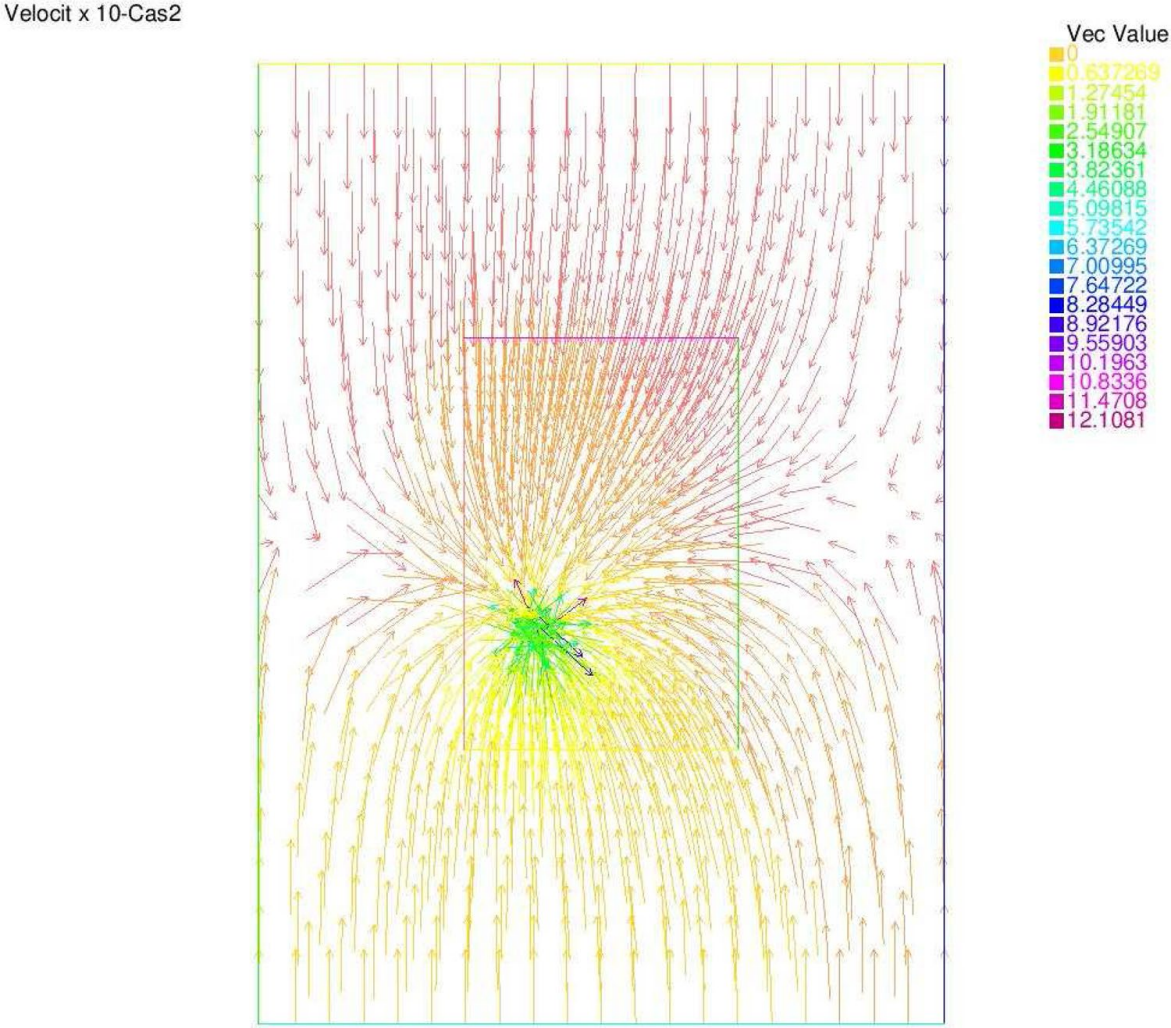
Velocity × 10—Case 2.

The reconstructed hydraulic head and its flux are given in Fig. 5, while the concentration are depicted in Fig. 6. Figure 7 describes the flow velocity of this second example. In Fig. 12 are shown the isovalues of the pollutant concentrations in the aquifer at the final time.

### Third example

It’s denoted by *Case 3* in the sequel. Here we consider a single point source with location: *S*(2.3, 1.2) and $Q=-0.0003$. We take $T=0.0001$, $h=3$ on $\Gamma _s$ and $h=0$ on $\Gamma _{\ell }$.

**Figure 8:**
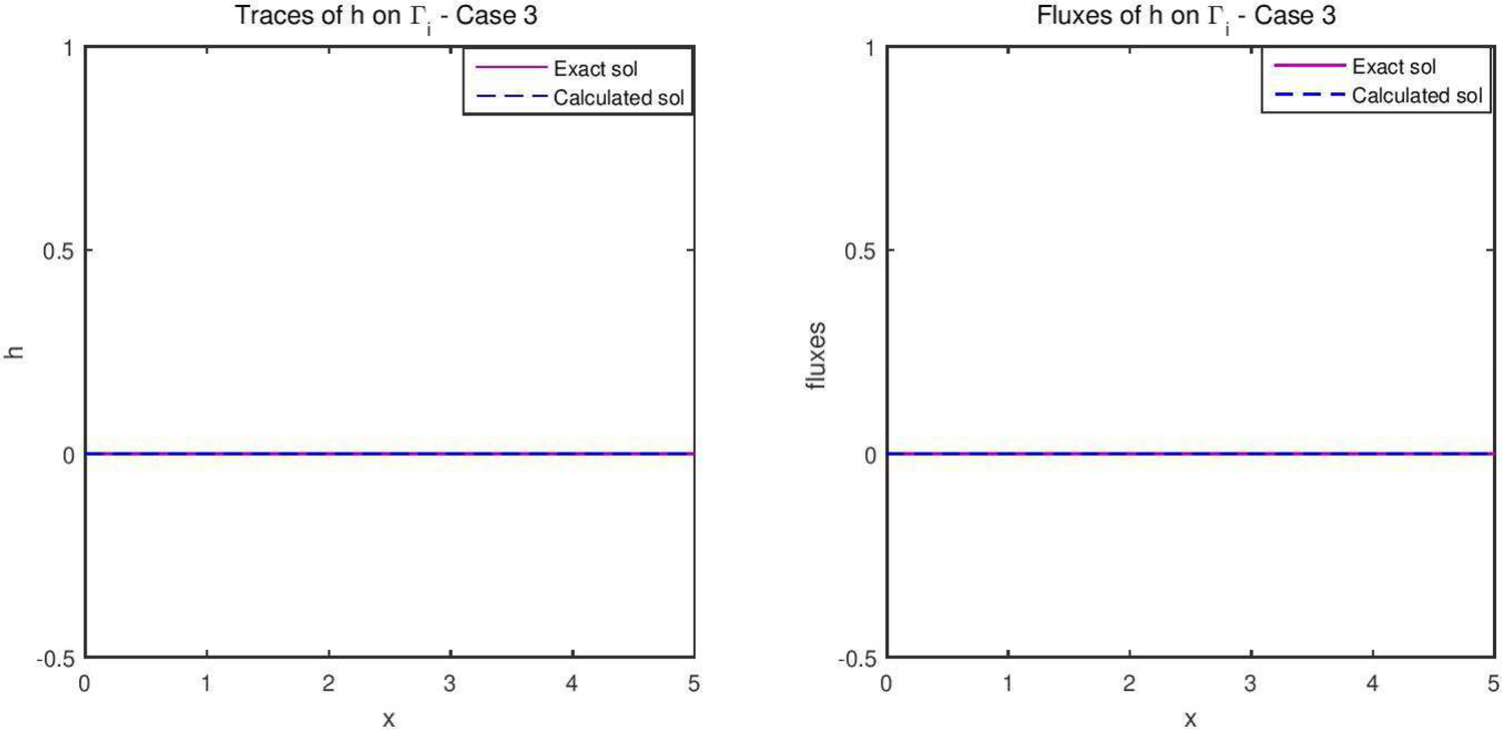
The reconstructed flow—Case 3.

**Figure 9:**
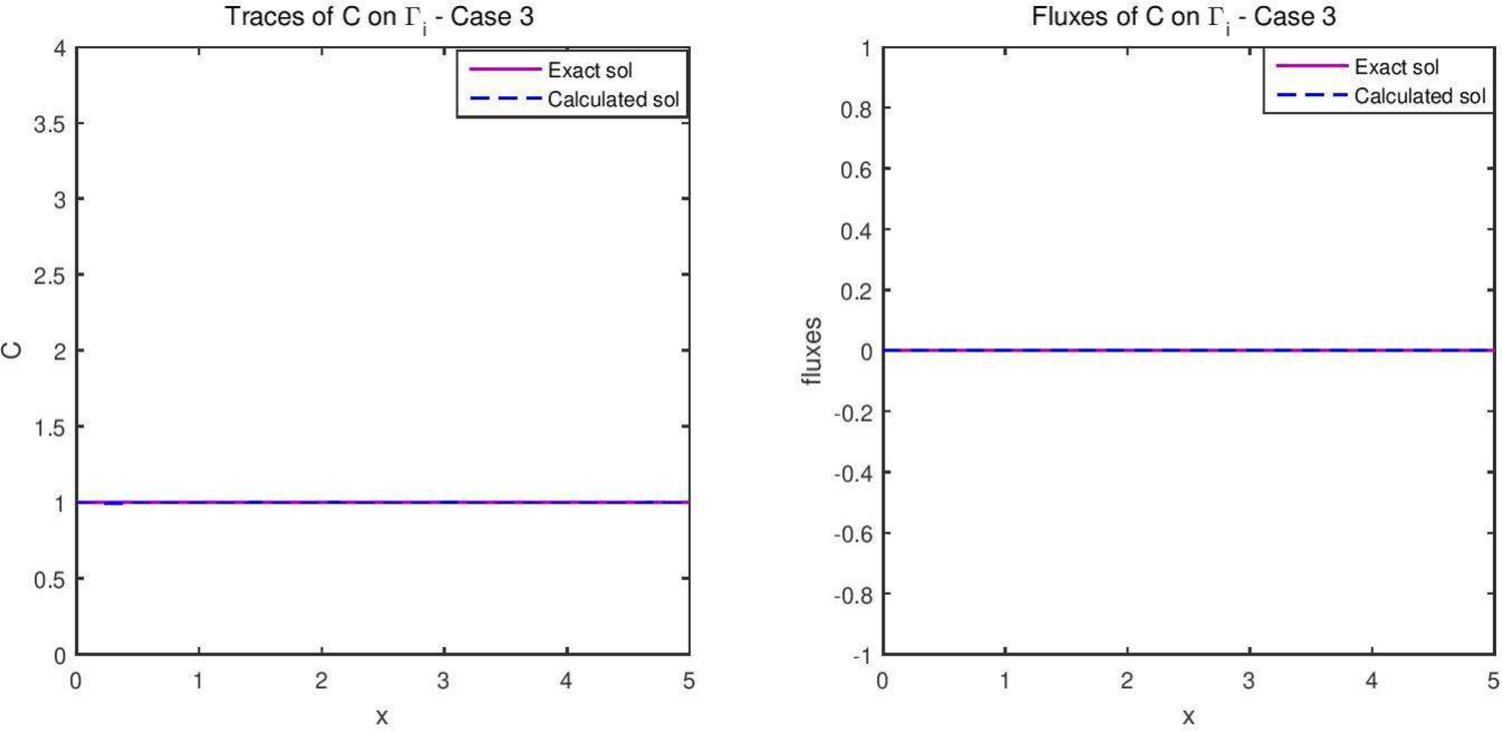
The reconstructed concentration—Case 3.

**Figure 10:**
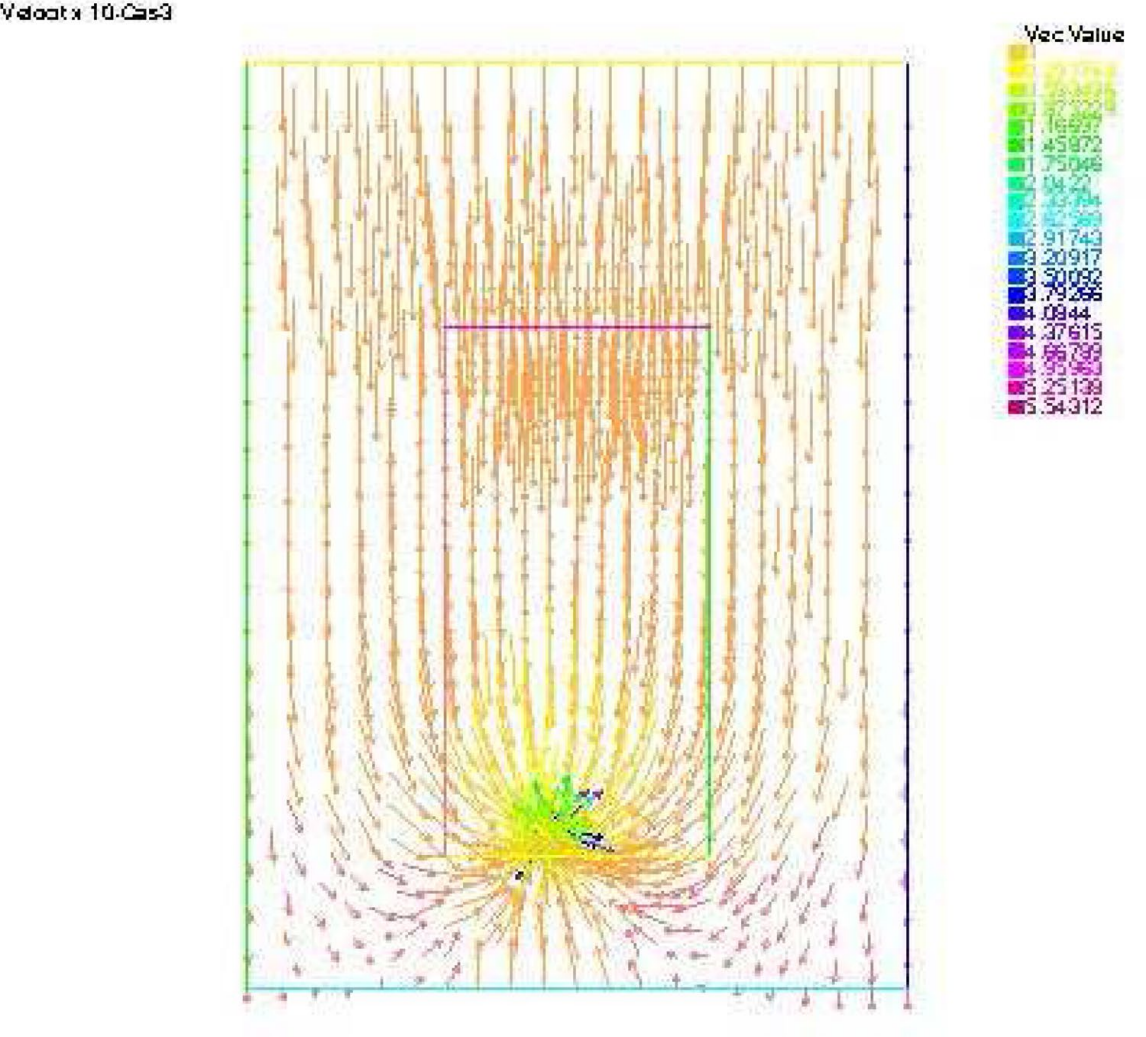
Velocity x 1e4—Case 3.

The reconstructed hydraulic head and the flux are given in Fig. 8, while the concentration is represented in Fig. 9. The flow velocity of this final example is given in Fig. 10.

## Discussion

**Figure 11:**
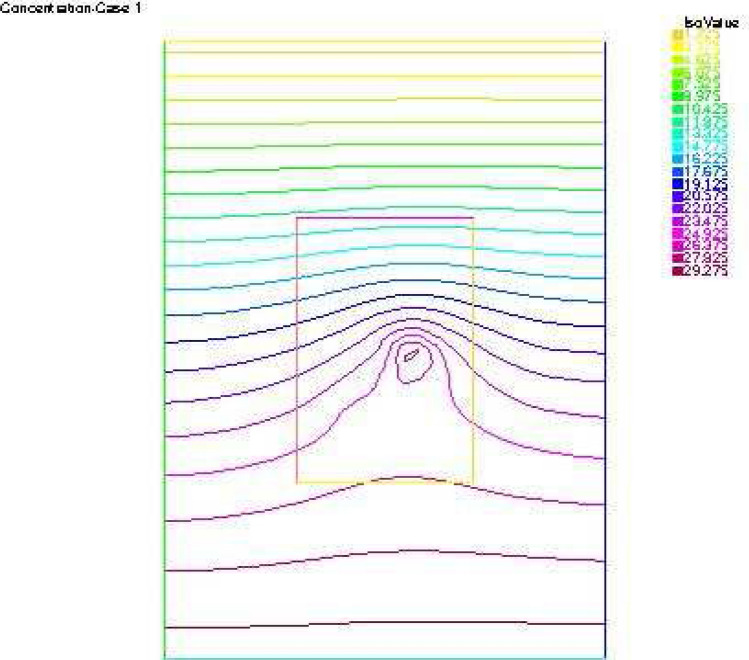
Pollutant concentrations—Case 1.

**Figure 12:**
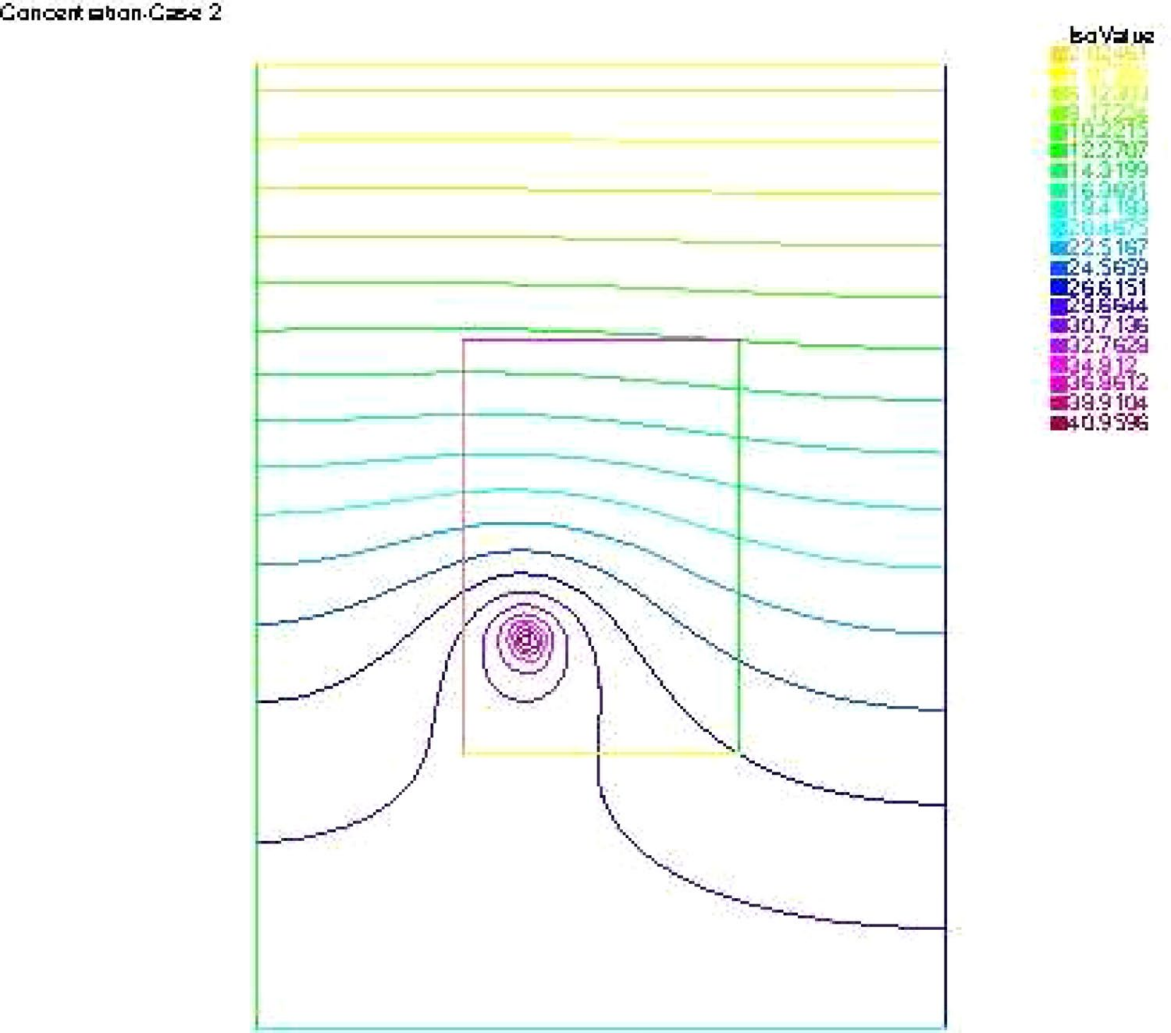
Pollutant concentrations—Case 2.

Synthetic data are used in the three simulated cases. Boundary conditions and pumping well flows are specifically chosen so that polluted water will invade the aquifer In all three cases, the hydraulic head and water flow are given on the upstream side while the pollutant concentration is imposed on the other side. Missing data on either boundary are reconstructed by the numerical model and compared to the exact values in Figs. 2, 3 for case 1, Figs. 5, 6 for case 2 and Figs. 8, 9 for case 3. For all cases, the boundary values are correctly identified. In Figs. 4 and 7, the velocity fields are shown for Case 1 and Case 2 respectively. It is pointed out that in both cases, the velocity vectors converge towards the wells, they cross the domain boundary on the polluted side, showing that there is intrusion of polluted water into the aquifer. Since the pumping fluxes from the wells are larger in case 2, the velocities are therefore larger in this case. The isovalues of the pollutant concentrations in the aquifer at the final time (Figs. 11 and 12) are concentric curves around the well. They show the intrusion of the pollution and its intensification near the well. The third case is specifically simulated with a single off-center well, located near the polluted side, with a low pumping rate, to test the model’s ability to identify a mixed inflow and outflow boundary condition on $\Gamma _s$. Figure 10, shows that the velocity vectors on this boundary are directed toward the aquifer near the well and toward the polluted zone elsewhere. The three simulated cases showed that the numerical model based on the data completion method is able to find the missing boundary conditions for the water flow coupled with solute transport in an aquifer.

## Conclusion

The main goal of this work was the development of a numerical tool to study the pollution of an aquifer by the intrusion of polluted water from a neighboring aquifer located downstream of the studied one. For this purpose, an inverse procedure based on the data completion method was developed. The direct problem model solves the coupled transient problems of groundwater flow and solute transport. The inverse method was used to reconstruct the missing data at the domain boundaries, thus circumventing the lack of information and exploiting the existence of over-specified data on the other boundaries.

The numerical model is tested on three examples with synthetic data. In these examples, the aquifers are exploited by pumping wells. To create different situations, well positions and flow rates were varied. The computational results show that the missing boundary conditions are well identified, even in the case of a mixed inlet-outlet boundary. The model is therefore able to answer the questions of the aquifer manager: is there polluted water intrusion? If so, where does it occur? And what is its extent?

As we have proven the validity of the method used and the robustness of the numerical model, we plan to use this promising tool, in future work, on real aquifers to detect the intrusion of polluted water or to locate the intrusion of seawater in coastal aquifers. The challenge would be to deal with complex geometry and variable parameters. Professional code would then be used to solve the direct problem. The FEFLOW, MODFLOW and SUTRA models can be called from user-defined code or from a bash file. SUTRA has already been implemented in an inverse problem solving procedure by Riahi et al.^23^. We can therefore consider using them for the direct problem as suggested by Bouhlila and Hariga^14^.

The present work developed tool could also be used to test pollution remediation scenarios by taking advantage of the available data and the low computational cost of the inverse method, thus reducing costly in situ measurement campaigns.

## Data Availability

All data generated or analysed during this study are included in this published article.
